# Complementary use of stable isotopes and fatty acids for quantitative diet estimation of sympatric predators, the Antarctic pack-ice seals

**DOI:** 10.1007/s00442-021-05045-z

**Published:** 2021-10-09

**Authors:** A. I. Guerrero, A. Pinnock, J. Negrete, T. L. Rogers

**Affiliations:** 1 Centro de Investigación y Gestión de Recursos Naturales (CIGREN), Instituto de Biología, Facultad de Ciencias, Universidad de Valparaíso, Gran Bretaña 1111, Playa Ancha, Valparaíso, Chile; 2 Evolution and Ecology Research Centre, School of Biological, Earth and Environmental Sciences, University of New South Wales, Sydney, 2052 Australia; 3 Instituto Antártico Argentino, Cerrito 1248, C10140 AAZ Buenos Aires, Argentina; 4 Centre for Marine Science and Innovation, School of Biological, Earth and Environmental Sciences, University of New South Wales, Sydney, 2052 Australia

**Keywords:** Biochemical tracer, Blubber, Marine mammal, Pinniped, Trophic marker

## Abstract

The quantitative use of stable isotopes (SIs) for trophic studies has seen a rapid growth whereas fatty acid (FA) studies remain mostly qualitative. We apply the Bayesian tool MixSIAR to both SI and FA data to estimate the diet of three sympatric predators: the crabeater (*Lobodon carcinophaga*), Weddell (*Leptonychotes weddellii*) and leopard seal (*Hydrurga leptonyx*). We used SI data of their vibrissae and FA data of their outer blubber to produce comparable diet estimates for the same individuals. Both SI and FA models predicted the same main diet components, although the predicted proportions differed. For the crabeater seal, both methods identified krill, *Euphausia superba*, as the main, and almost exclusive, food item, although the FA model estimated a slightly lower proportion, potentially due to the low lipid content of krill compared to the fish species used in the model. For the Weddell seal the FA model identified the fish *Pleuragramma antarcticum* as the most important prey, whereas the SI model was not able to distinguish among prey species, identifying a ‘fish-squid’ group as the main diet component. For the leopard seal, both models identified krill as the main contributor; however, the predicted proportions for the secondary sources differed. Although vibrissae and outer blubber may not represent the same timeframe, the use of MixSIAR with FA data provides diet estimates comparable to those obtained with SI data, thus, both approaches were complimentary. The use of both biotracers offers a feasible option to study diets of wild animals in a quantitative manner.

**Supplementary Information:**

The online version contains supplementary material available at 10.1007/s00442-021-05045-z.

## Introduction

The study of marine predators is particularly challenging due to low encounter rates and laborious handling of wild animals. Until a few decades ago, our understanding of their ecology consisted of limited observations as they came ashore or near the surface. Traditionally, we have relied on methods such as stomach and scat content analyses to study their foraging ecology (Hall-Aspland and Rogers 2004; Acevedo et al. 2015). Although these techniques provide valuable information, they are usually based on few and recent feeding events, and have known biases related to identification of prey items and differential digestion rates (Gales and Cheal 1992), making it difficult to answer complex ecological questions.

The implementation of new methods such as satellite transmitters and biochemical analyses has allowed to substantially broaden our knowledge of the behaviour and foraging ecology of marine predators (Iverson et al. 1997). Biochemical methods, such as fatty acid (FA) and stable isotope (SI) analyses, can reconstruct diets by overcoming the caveats of the traditional techniques. These two biochemical methods are based on the principle that an animal’s diet is reflected in the patterns of FAs and SIs of their tissues (Hooker et al. 2001; Guerrero et al. 2020). Many FAs, particularly polyunsaturated FAs, can only be synthesized at low trophic levels and are transferred to higher trophic levels with minimal modification (Iverson 1993) and thus, can be used to distinguish dietary preferences (Dalsgaard et al. 2003; Guerrero et al. 2020). Similarly, the isotopic composition of an animal’s tissues is correlated with those of its prey items and change in a predictable way between trophic levels (Gannes et al. 1997; Hückstädt et al. 2012). Thus, these biochemical compounds have the potential to be used as biological tracers (“biotracers”).

Although these two methods have been widely used as dietary predictors, there has been controversy regarding their actual accuracy in predicting diet when used alone (Gannes et al. 1997; Grahl-Nielsen et al. 2011; Rosen and Tollit 2012). Limitations to the use of FAs as biotracers include the difficulty of tracing FAs in upper trophic levels, because they typically originate from a variety of sources and thus they become obscured as they travel up the food web (Dalsgaard et al. 2003; Wheatley et al. 2007). In addition, many of these FAs have specific physiological functions (Alonzo et al. 2005); therefore, their abundance in the tissues can vary according to the animal’s physiological state. Similarly, the use of SIs has constraints such as the limited number of variables (e.g. carbon and nitrogen) used for dietary predictions, which limits the applicability of some statistical models as usually only a corresponding number of prey items can be incorporated into the analysis (Bromaghin 2017). It is usual that several prey species share similar SI values and therefore they need to be combined into a single prey group (e.g. Hückstädt et al. 2012; Goetz et al. 2016; Botta et al. 2018); making it difficult to obtain a good taxonomic resolution of the predator’s diet (Guerrero and Rogers 2020). Considering these limitations, the combined use of these two biotracers has been proposed as a more accurate technique to estimate diet composition (Dalsgaard et al. 2003).

Despite having a predominantly qualitative focus, trophic studies based on FA data have enabled the understanding of energy flow within food webs (e.g. Beck et al. 2007; McMeans et al. 2012; Guerrero et al. 2016). More recently, efforts have concentrated on developing robust biotracer-based quantitative methods to obtain accurate estimates of a predator’s diet (Goetsch et al. 2018).

The Bayesian mixing model tool MixSIAR (Stock et al. 2018), although originally developed for SI data, can be applied to other mixing processes, as it estimates the contribution of different sources (prey) to a mixture (consumer). However, its use with FA data has received less attention compared to SI data. Guerrero and Rogers (2020) tested the performance of MixSIAR with FA data for diet estimation of captive animals undergoing feeding experiments, including different consumer species such as fish, birds and marine mammals, with different diet compositions. MixSIAR accurately estimated consumers’ main dietary items, and identified shifts in diet and absent prey (Guerrero and Rogers 2020). To date, however, MixSIAR has not been used to study the diet of wild animals based on their FAs. Here, we implement the MixSIAR approach with paired FA and SI data from wild animals. The aim of this study is to evaluate how FAs and SIs perform relative to each other in predicting diet composition of three predators within the Antarctic ecosystem.

We estimated the diet composition in three sympatric Antarctic pack-ice seals: the leopard (*Hydrurga leptonyx*), crabeater (*Lobodon carcinophaga*) and Weddell seal (*Leptonychotes weddellii*), using FAs from their outer blubber, and SIs from their whiskers. These top predators inhabit the western Antarctic region, that is being dramatically affected by climate change, which is in turn shaping ecosystem dynamics (Clarke et al. 2007). Predators can evidence changes in the distribution, abundance, and composition of the prey community (Hazen et al. 2019). Quantitative data of their foraging behaviour is, therefore, important to understand and monitor ecosystem status, and predict potential changes (Fleming et al. 2016).

Leopard seals feed on a variety of prey at different trophic levels, such as krill, fish, penguins and other seals (Siniff and Stone 1985; Green and Williams 1986; Rogers and Bryden 1995; Hall-Aspland and Rogers 2004; Casaux et al. 2009; Botta et al. 2018; Krause et al. 2020); whereas crabeater seals specialise almost exclusively on krill (Laws 1977; Hückstädt et al. 2012), and Weddell seals prey mostly on fish, but also on cephalopods (Burns et al. 1998; Zhao et al. 2004; Casaux et al. 2011; Acevedo et al. 2015; Goetz et al. 2016). Thus, we evaluated how the combined use of FAs and SIs performs to estimate the relative dietary composition of predators with different foraging strategies.

## Methods

### Sample collection of seals

During the austral summer of 2015, 21 crabeater seals (10 adult males, 9 adult females, and 2 juvenile females), 18 Weddell seals (6 adult males and 12 adult females) and 13 leopard seals (10 adult males, 2 adult females, and 1 juvenile male) were sampled while hauled out on sea ice off the Danco Coast, Western Antarctic Peninsula (64°09′ S 60°57′ W). Seals were immobilised using Zoletil (tiletamine/zolazepam, Virbac) delivered via a Tele-inject air gun darting system (Higgins et al. 2002). All applicable institutional guidelines for the care and use of animals were followed. The immobilisation and sampling of seals in the Antarctic Specially Protected Area No. 134 was approved by the Dirección Nacional del Antártico, Buenos Aires, Argentina; and performed according to the SCAR code of conduct for animal experiments under UNSW Animal Care and Ethics Committee (Approval 15/55A). Following immobilisation, one whisker was plucked and an 8 mm diameter biopsy sample collected from the mid-dorsal region to a depth that incorporated the outer blubber and the skin. Skin and outer blubber layer were separated and stored in airtight vials at − 20 °C.

### Sample and data collection of prey

Antarctic krill, *Euphausia superba*, samples were obtained opportunistically from stomachs of deceased leopard seals in 2008; and from stomachs of gentoo, *Pygoscelis papua*, and chinstrap, *P. antarcticus*, penguins in 2015. Muscle samples of adult gentoo penguins were collected opportunistically in 2008 and 2012, in the same study area, from fresh carcasses found in the vicinities of penguin colonies. All these samples were analysed for FAs only, following the corresponding procedure described further below.

SI values and other FA values for potential prey were obtained from the published literature (see Table 1). We used the same prey species for both FA and SI models, except for the cephalopod species, where we used *Moroteuthis ingens* for the FA model and *Pareledone sp* for the SI model*.* For the Antarctic fur seal, *Arctocephalus gazella,* we used milk FAs and SIs, as muscle or blubber samples were not available. Table 2 shows the prey species included in the models to estimate the diet of each seal species.

**Table 1:** Literature sources for each prey species used in diet estimation models

Species	Citation	
	Fatty acid data	Stable isotope data
*Euphausia superba*	Fricke et al. (1984), Phleger et al. (2002), this study	Polito and Goebel (2010), Polito et al. (2011)
*Electrona carlsbergi*	Stowasser et al. (2009)	Polito and Goebel (2010)
*Electrona antarctica*	Stowasser et al. (2009)	Polito and Goebel (2010)
*Pleuragramma antarcticum*	Hagen et al. (2000)	Polito et al. (2011)
*Arctocephalus gazella*	Iverson et al. (1997)	Polito and Goebel (2010)
*Pareledone *sp.	–	Mincks et al. (2008)
*Pygoscelis papua*	This study	Polito et al. (2011)
*Moroteuthis ingens*	Phillips et al. (2001)	–
*Gymnoscopelus nicholsi*	Stowasser et al. (2009)	Stowasser et al. (2012)

**Table 2:** Source (prey) species used to estimate the diet of each consumer (seal)

Consumer	Prey taxon	Sources	
		Fatty acid model	Stable isotope model
Crabeater seals	Krill	*E. superba*	*E. superba*
	Fish	*E. carlsbergi*	*E. carlsbergi*
	Fish	*E. antarctica*	Pa_Ea^a^
	Fish	*P. antarcticum*	
Leopard seals	Seal	*A. gazella*	*A. gazella*
	Krill	*E. superba*	*E. superba*
	Fish	*P. antarcticum*	*P. antarcticum*
	Penguin	*P. papua*	*P. papua*
	Cephalopod	*M. ingens*	*Pareledone sp.*
Weddell seals	Krill	*E. superba*	*E. superba*
	Fish	*G. nicholsi*	*G. nicholsi*
	Fish	*E. antarctica*	Pa_Ea_P^a^
	Fish	*P. antarcticum*	
	Cephalopod	*M. ingens*	

The group “Pa_Ea” correspond to the combined isotopic values of the fish *P. antarcticum* and *E. antarctica,* and the group “Pa_Ea_P” is the combination of these two fish species and the squid *Pareledone *sp

^a^Group of combined species

### Fatty acid analysis

Total lipid of seal blubber, whole krill, and penguin muscle, was extracted following a modified Folch et al. (1957) method (Budge et al. 2006). Briefly, approximately 0.2–0.5 g of tissue was extracted using 2:1 chloroform: methanol with 0.01% of butylated hidroxytoluene, washed in a salt solution, centrifuged, dried over anhydrous sodium sulphate and evaporated under nitrogen. FA methyl esters were prepared using H_2_SO_4_ in methanol and then extracted into hexane (50 mg/ml).

Gas chromatography analyses were performed with Agilent 7890A Series GC System (Agilent Technologies, USA) equipped with a flame ionization detector, as described in Guerrero and Rogers (2017). Identification of FAs and isomers was conducted using known standard mixtures (Nu Check Prep., Elysian, MN, USA). Once FAs were identified, their concentrations were converted to percentage contributions of the total FAs.

### Stable isotope analysis

Prior to analysis, whiskers were cleaned in chloroform:methanol (2:1 v/v) in an ultrasonic bath and allowed to air dry for at least 48 h. Whiskers were sectioned into 2 mm subsamples of 0.2 ± 0.1 mg and placed into tin capsules for analysis. For diet estimation using MixSIAR, we used a mean isotope value of the whole whisker for each consumer.

Whisker samples were analysed using a Flash 2000 organic elemental analyser (ThermoFisher Scientific) interfaced to a Delta V Advantage Isotope Ratio Mass Spectrometer via a ConFlo IV interface (Bioanalytical Mass Spectrometry Facility, UNSW, Australia).

Stable carbon and nitrogen-isotope ratios are reported as δ^13^C and δ^15^N, respectively, in parts per thousand (‰):

$${\delta }^{13}\mathrm{C} \mathrm\;{ \text{or} }\; {\delta }^{15}\mathrm{N}\left(\permil \right)={[(R}_{\mathrm{sample}}/{R}_{\mathrm{standard}})-1]\times 1000,$$

where *R* is the ratio of the heavier isotope to the lighter isotope (^13^C/^12^C or ^15^N/^14^N). The standards correspond to Vienna-Peedee belemnite (V-PDB) for carbon, and atmospheric nitrogen for nitrogen, reference standard of nitrogen and carbon, Glutamic 40 and Glutamic 41, were included after every 10 samples to account for machine drift.

### Diet estimation

Three datasets are necessary to estimate diet using Bayesian mixing models: signatures of consumers (predator), signatures of sources (prey) and values of trophic discrimination (differences in biotracer values between prey and predator). We used the discrimination values 2.2 ± 0.7‰ for δ^13^C and 3.5 ± 0.6‰ for δ^15^N, obtained by Newsome et al. (2010) for the sea otter, *Enhydra lutris*.

The equivalent to discrimination values for FA data are calibration coefficients, which are used to account for selective FA metabolism and hence changes in the predator’s FA proportions relative to diet consumed (Iverson et al. 2004; Guerrero and Rogers 2020). We used the calibration coefficients derived from captive harbour seals, *Phoca vitulina*, fed herring for over a year (Rosen and Tollit 2012), and applied them to our three study species. Prior to the Bayesian mixing model analysis, the FA signatures of prey items were multiplied by the calibration coefficients of harbour seals; thus, taking prey signatures to the predator space (Bromaghin 2017; Guerrero and Rogers 2020). This was done prior to analysis since MixSIAR treats discrimination as additive values, whereas FA calibration coefficients are multiplicative values. Therefore, as the prey values already accounted for the ‘enrichment’ in FA proportions, we then set discrimination values to zero.

For diet estimation analyses, we used dietary and extended dietary FAs, according to Iverson et al. (2004), but discarded those FAs with high calibration coefficients (> 3), since they indicate greater influence of consumer metabolism or preferential accrual rather than a reflection of diet (Guerrero and Rogers 2020). Thus, the number of FAs used for analyses, for each consumer species, was 12.

MixSIAR requires sources values to be statistically different. Therefore, prior to analysis, we tested the differences in biotracer values among sources both visually and statistically. For each FA model, we conducted a Non-metric multi-dimensional scaling (NMDS) analysis to visually assess differences among sources. For each SI model, we produced a biplot to assess visual differences among sources. For statistical confirmation, we conducted pairwise multilevel comparisons in the R package “pairwiseAdonis” (Martinez Arbizu 2017), which is based on the *adonis* function of the R package “vegan” (Oksanen et al. 2018) that implements a multivariate analysis of variances using distance matrices. When sources were not statistically different from each other, they were combined into a single group based on the similarity of their biotracer values.

We used the Bayesian mixing tool MixSIAR (Stock et al. 2018) for both FA and SI data. To run the model, biotracer values for each consumer were input as raw data. Biotracer values for each source were input into the model as the mean and standard deviation. For both FA and SI data, we used non-informative priors. We used multiplicative process error structure (Stock and Semmens 2016) and model convergence was assessed via Gelman–Rubin and Geweke diagnostics (Geweke 1991; Gelman et al. 2014).

Data analyses were conducted using RStudio software, version 1.1.456 (RStudio Team 2016). Posterior distributions obtained from the MixSIAR analyses are expressed as median (and range).

## Results

**Fig. 1:**
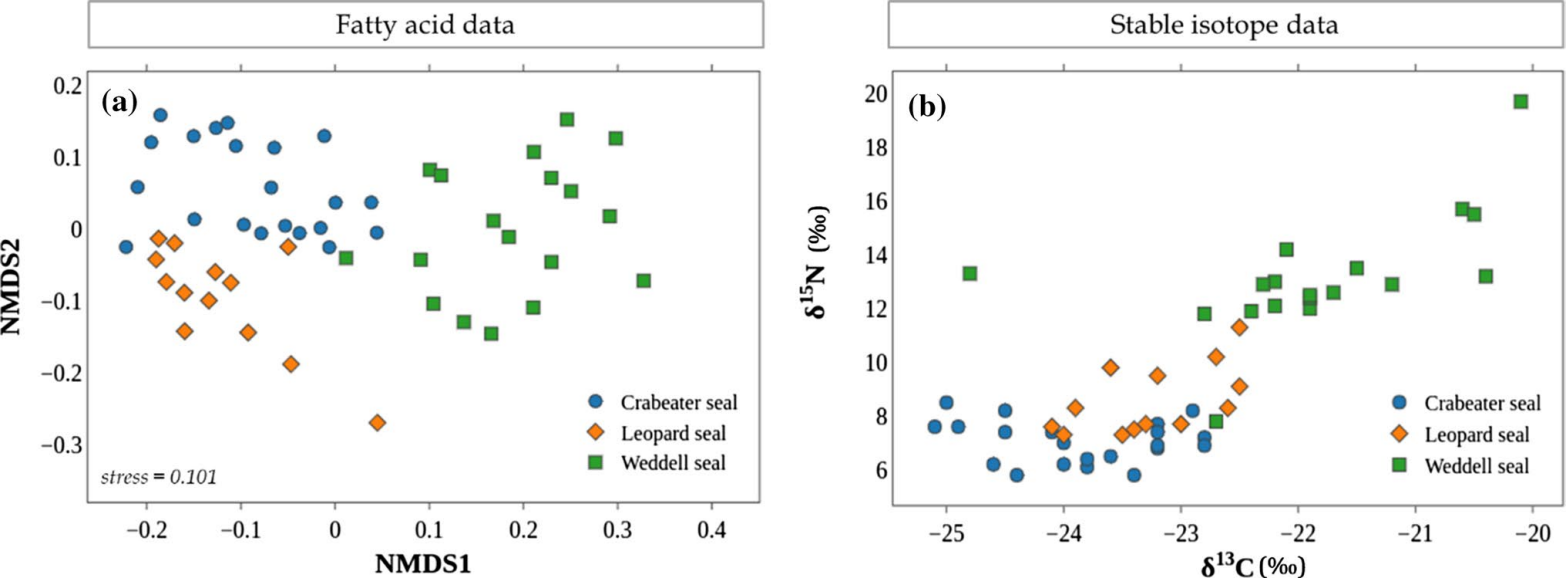
Biplots for biotracer values of crabeater (*n* = 21), Weddell (*n* = 18) and leopard seals (*n* = 13): **a** non-metric multi-dimensional scaling (NMDS) plot of the blubber FA profiles, and **b** biplot of the isotopic values of whiskers

The NMDS analysis of individual animals showed three distinct groups that represented the three species. Leopard and crabeater seals were closer to each other in the plot, indicating more similarity in their FA profiles (Fig. 1a). Similarly, the isoplot (Fig. 1b) showed the three species as separate groups, although there was overlap between the crabeater and leopard seals. Crabeater seals had the lowest δ^13^C and δ^15^N values whereas Weddell seals had the highest isotopic values, indicating higher trophic position.

### Diet estimation for crabeater seals

**Fig. 2:**
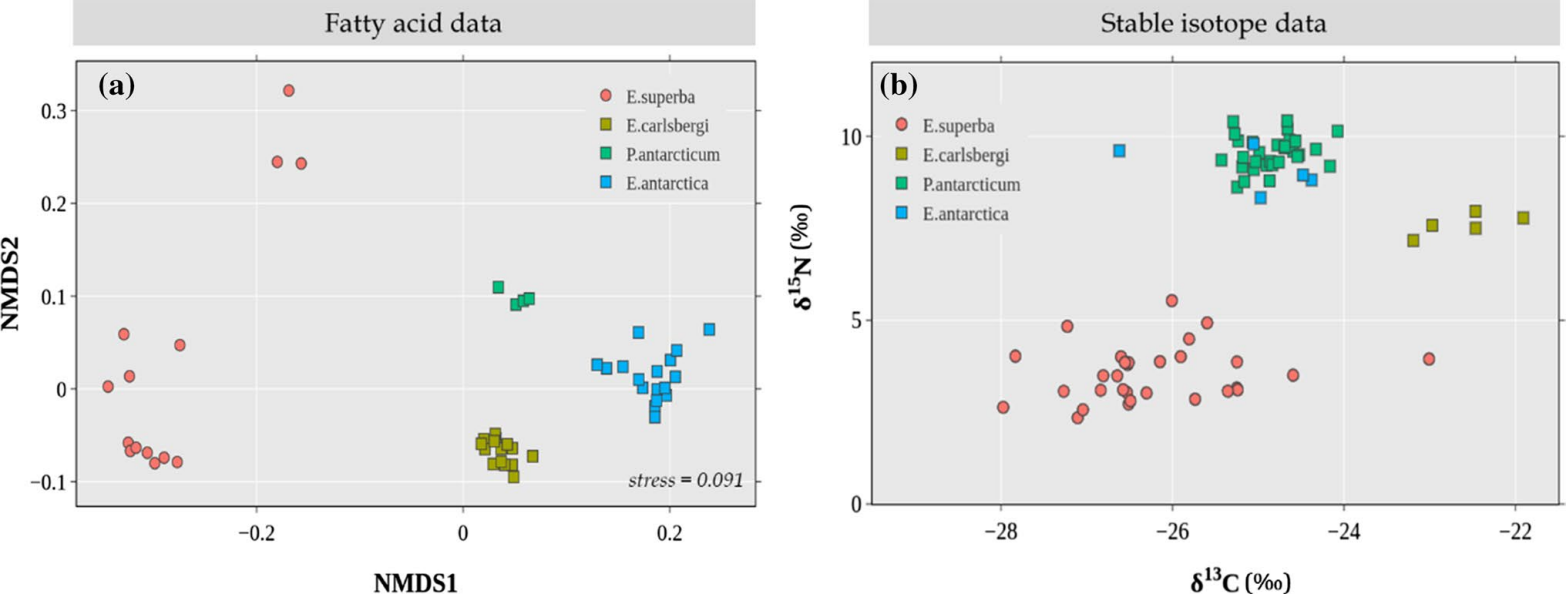
Biplots for biotracer values of source species used to estimate the diet of crabeater seals: **a** Non-metric multi-dimensional scaling (NMDS) plot of their FA profiles, and **b** biplot of their isotopic values. Square symbols represent fish species, whereas the circle symbol is krill. Because of the isotopic similarity between the fish species *P. antarcticum* and *E. antarctica*, they were later combined into a group named *Pa_Ea* for diet estimation

Prey species used to estimate the diet of crabeater seals via FAs were statistically different from each other (*F*_3_ = 90.78, *P* = 0.001, Fig. 2a), therefore, all four species were input into the model. For SI-based estimations, although the groups were different (*F*_3_ = 209.44, *P* = 0.001), pairwise comparisons revealed that the fish species *P. antarcticum* and *E. antarctica* were not statistically different (*F*_1_ = 1.01, adjusted* P* = 1.00, Fig. 2b), hence, they were combined into a single group named “Pa_Ea” (Table 2).

**Fig. 3:**
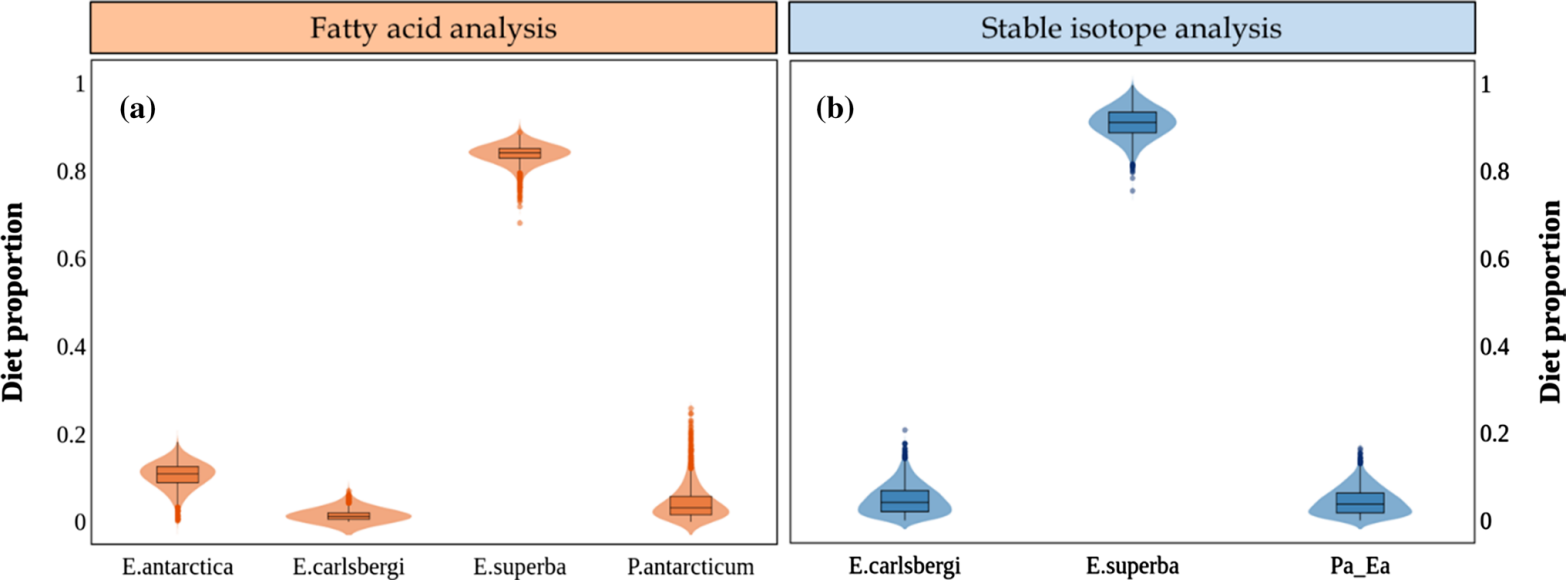
Diet estimation for crabeater seals (*n* = 21) using the Bayesian mixing model MixSIAR, based on **a** fatty acid or **b** stable isotope data. The prey “Pa_Ea” corresponds to the isotopic values of *P. antarcticum* and *E. antarctica* combined into a single group, due to their isotopic similarity

Both biotracers identified krill, *E. superba*, as the main component of the diet of crabeater seals. The FA model estimated that *E. superba* contributed 84% to the diet, whereas the SI model determined that *E. superba* contributed 91% (Table 2, Fig. 3). The FA model estimated that *E. antarctica* was the most important fish species, with 11% of contribution, however the SI model attributed almost equal importance to all fish species/groups (4% each).

### Diet estimation for Weddell seals

**Fig. 4:**
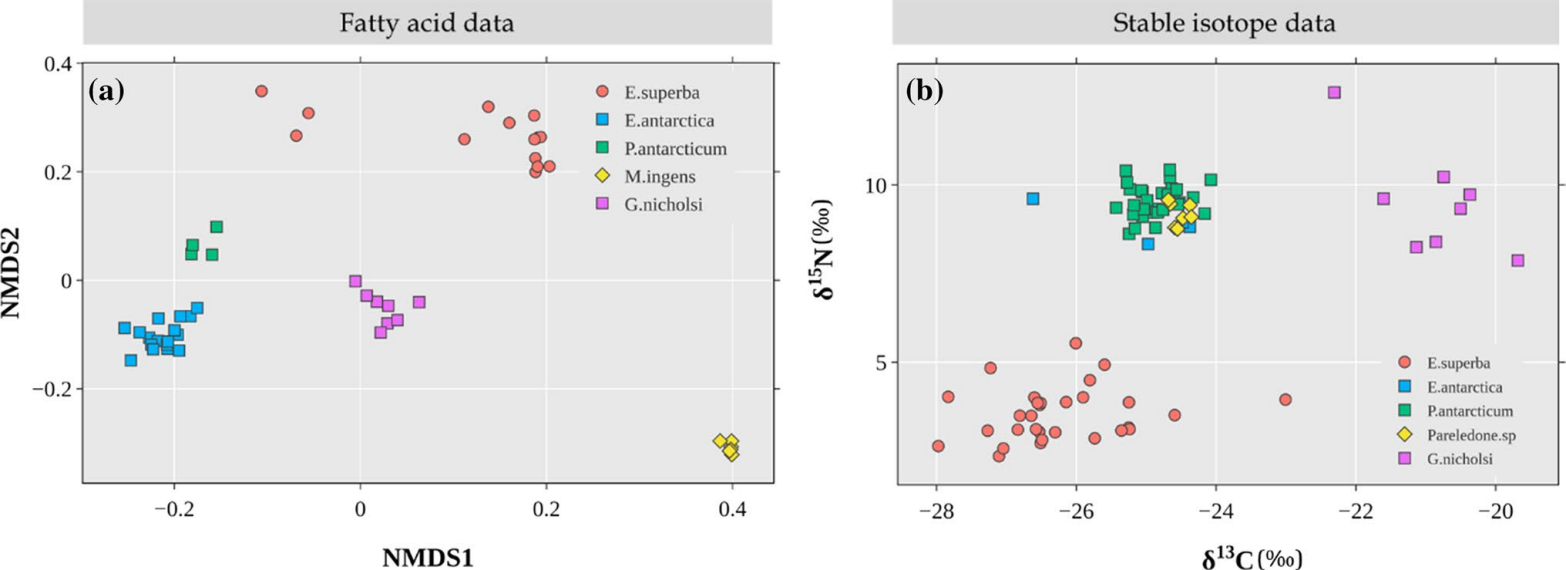
Biplots for biotracer values of source species used to estimate the diet of Weddell seals: **a** non-metric multi-dimensional scaling (NMDS) plot of their FA profiles, and **b** biplot of their isotopic values. Square symbols represent fish species, the diamond symbol represent cephalopod species, whereas the circle symbol is krill. The fish species *P. antarcticum* and *E. antarctica*, and the cephalopod *Pareledone* sp., are isotopically very similar; therefore they were combined into a single group named *Pa_Ea_P* for diet estimation

Due to lack of biotracer data, we used the cephalopod *M. ingens* for FA models and *Pareledone *sp. for SI models. As the sources used for diet estimation of Weddell seals had different FA profiles (*F*_4_ = 96.90, *P* = 0.001, Fig. 4a) we used all five source species in the FA model (Table 2). Based on SI values, the groups were different overall (*F*_4_ = 183.56, *P* = 0.001, Fig. 4b), although pairwise comparisons show that there were no differences between the fish species *P. antarcticum* and *E. antarctica* (*F*_1_ = 1.01, adjusted* P* = 1.00), between *E. antarctica* and the cephalopod *Pareledone *sp. (*F*_1_ = 1.27, adjusted* P* = 1.00), and between *P. antarcticum* and *Pareledone *sp. (*F*_1_ = 4.95, adjusted* P* = 0.20), therefore these three species were combined into a group, named “Pa_Ea_P” (Table 2).

**Fig. 5:**
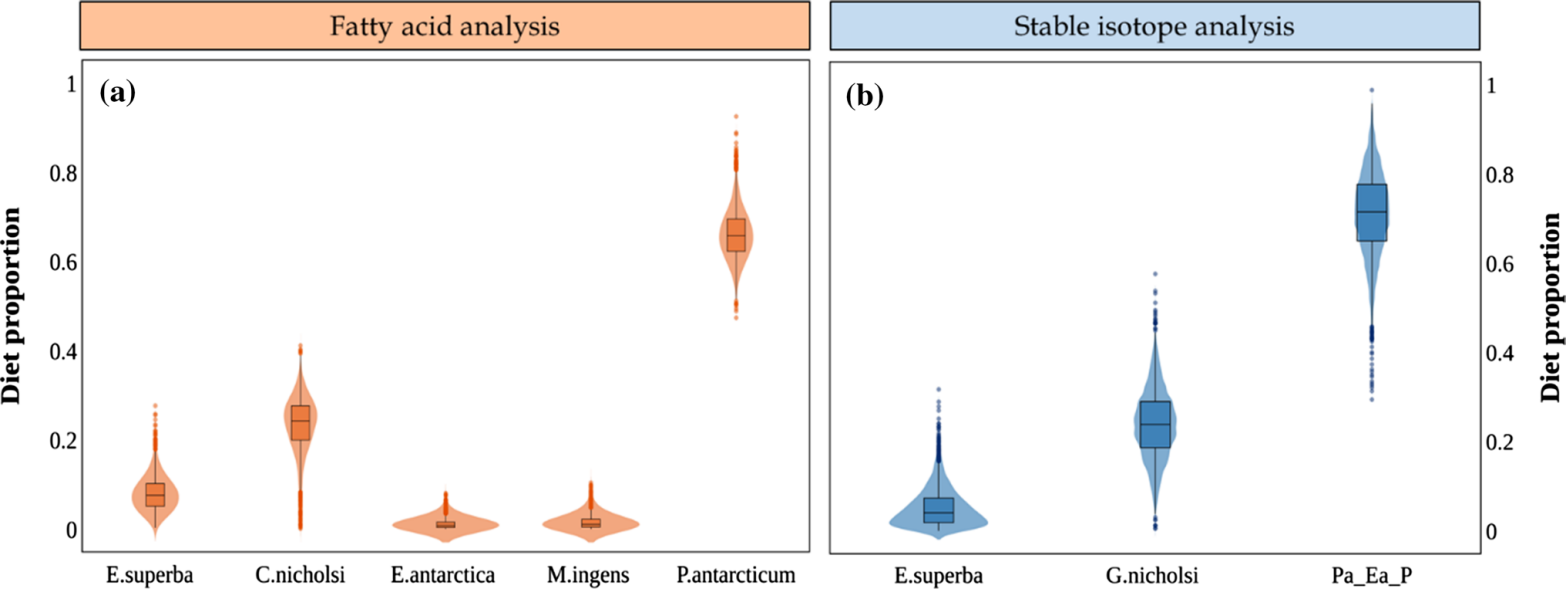
Diet estimation for Weddell seals (*n* = 18) using the Bayesian mixing model MixSIAR, based on **a** fatty acid or **b** stable isotope data. The prey “Pa_Ea_P” corresponds to the isotopic values of *P. antarcticum*, *E. antarctica* and *Pareledone *sp, combined into a single group, due to their isotopic similarity

Based on FA data, MixSIAR estimated that 66% of the diet of Weddell seals was composed of *P. antarcticum*, followed by 24% of *G.nicholsi*, 8% of *E. superba*, whereas *E. antarctica* and the squid *M. ingens* were negligible (Table 3, Fig. 5a). Based on SI data, the estimated main component of the diet of Weddell seals was the group Pa_Ea_P, with 72%, followed by *G. nicholsi* and *E. superba*, with 24% and 4%, respectively (Table 3, Fig. 5b).

**Table 3:** Proportions of diet estimated for crabeater seals, by MixSIAR using fatty acid or stable isotope data

Sources	Diet estimation for crabeater seals	
	Fatty acids	Stable isotopes
*E. superba*	0.840 (0.799–0.867)	0.911 (0.836–0.972)
*E. carlsbergi*	0.012 (0.001–0.042)	0.041 (0.002–0.125)
*E. antarctica*	0.109 (0.042–0.150)	–
*P. antarcticum*	0.032 (0.002–0.145)	–
*Pa_Ea*	–	0.037 (0.002–0.110)

The group “Pa_Ea” corresponds to the combined isotope values of the fish *P. antarcticum* and *E. antarctica*

### Diet estimation for leopard seals

**Fig. 6:**
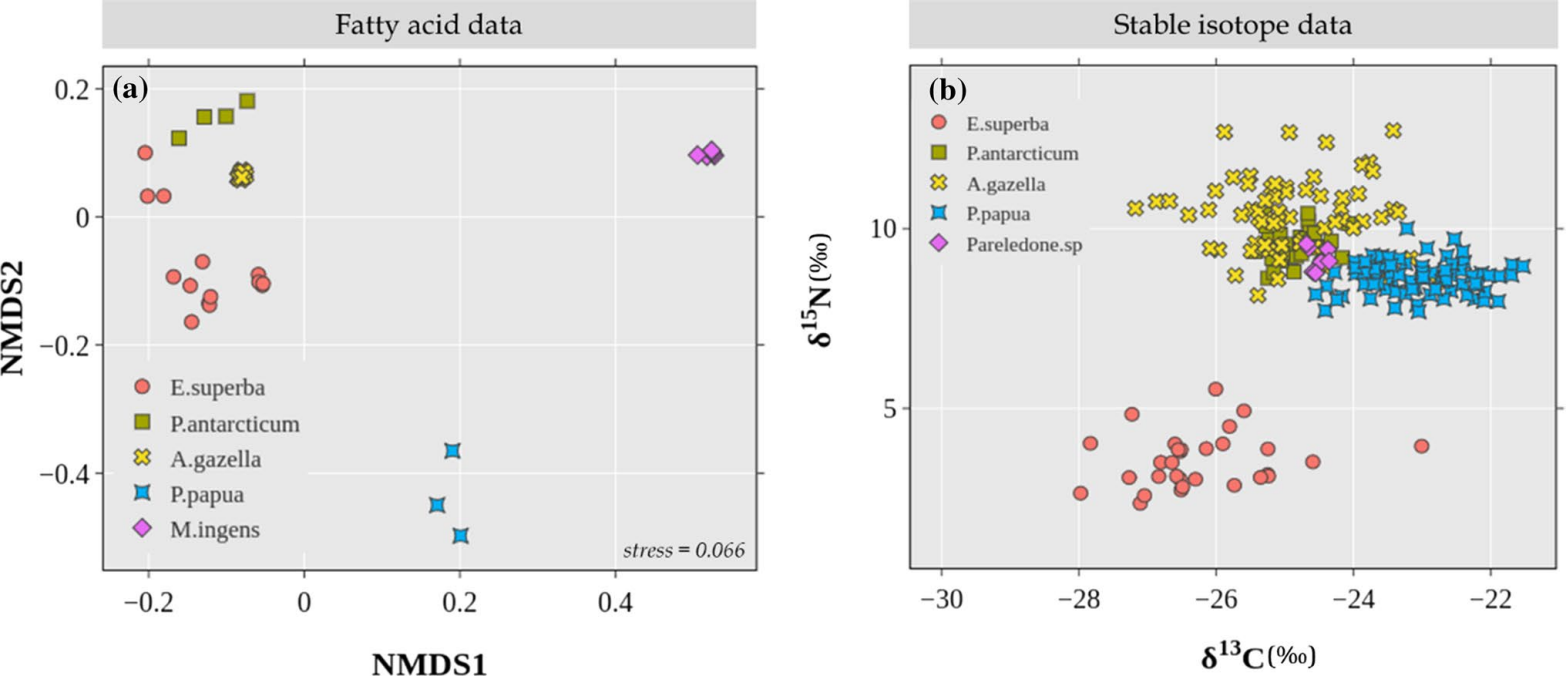
Biplots for biotracer values of source species used to estimate the diet of leopard seals: **a** non-metric multi-dimensional scaling (NMDS) plot of their FA profiles, and **b** biplot of their isotopic values

*Pareledone *sp. was used for the SI model and *M. ingens* for the FA model. Source species had different SI (*F*_4_ = 308.04, *P* = 0.001, Fig. 6a) and FA values (*F*_4_ = 96.52, *P* = 0.001, Fig, 6b), thus, all sources were used in the models (Table 2).

**Fig. 7:**
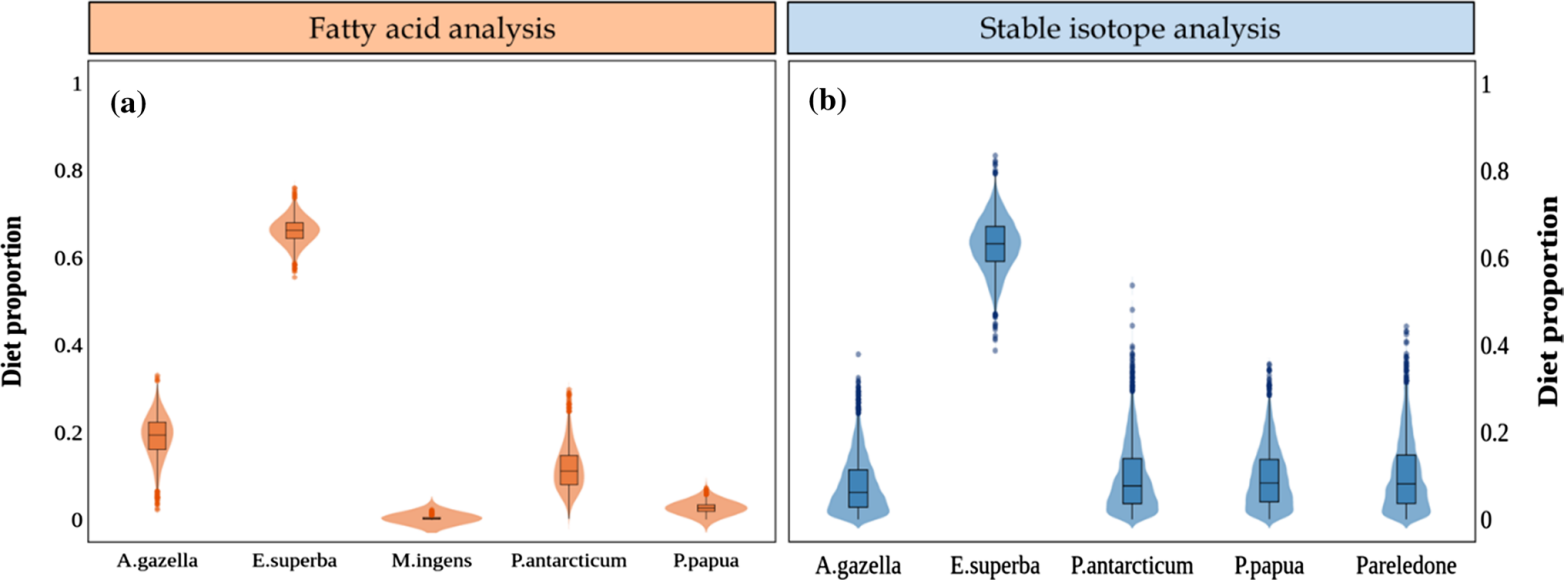
Diet estimation for leopard seals (*n* = 13) using the Bayesian mixing model MixSIAR, based on **a** fatty acid or **b** stable isotope data

Both FA and SI models identified *E. superba* as the main component of the leopard seal’s diet (Fig. 7). The model based on FA data estimated that *E. superba* was 66% of their diet whereas the SI model estimation was 63% (Table 4). The FA model determined that the second most important source, with 19% contribution, was the Antarctic fur seal, *A. gazella*, followed by an 11% contribution from the Antarctic silverfish, *P. antarcticum*, and 3% from the gentoo penguin, *P. papua*, whereas the cephalopod *M. ingens* was insignificant. Conversely, the SI model estimated the contribution of the four sources (*A. gazella*, *P. antarcticum*, *P. papua* and *M. ingens*) as almost equal (between 6% and 8%).

**Table 4:** Proportions of diet estimated for Weddell seals, using MixSIAR with fatty acid or stable isotope data

Sources	Diet estimation for Weddell seals	
	Fatty acids	Stable isotopes
*E. superba*	0.076 (0.038–0.164)	0.040 (0.002–0.138)
*E. antarctica*	0.008 (0.000–0.039)	–
*P. antarcticum*	0.657 (0.558–0.782)	–
*M. ingens*	0.011 (0.000–0.057)	–
*Pa_Ea_P*	–	0.715 (0.496–0.860)
*G. nicholsi*	0.242 (0.080–0.338)	0.238 (0.085–0.373)

The group “Pa_Ea_P” correspond to the combined isotope values of the fish *P. antarcticum* and *E. antarctica*, and the squid *Pareledone *sp

## Discussion

We show that models (using Bayesian tool MixSIAR) that use FA and SI data predict similar diets for three sympatric top predators with different foraging strategies. This approach, the combination of two different biotracers using the same quantitative statistical tool, has the potential to produce complementary diet estimates. To date, few studies have applied quantitative tools to both biotracers (e.g. Neubauer and Jensen 2015), and typically the complementary use of these two biotracers, FA and SI, has relied on the qualitative use of FA data (e.g. Hooker et al., 2001; Herman et al., 2005). FA data can be used quantitatively with a software tool broadly used with SI data. Recent application of MixSIAR to FA data has shown that this approach correctly identifies main diet components for captive animals with known diets (Guerrero and Rogers 2020) and herbivorous zooplankton using simulated FA data (Litmanen et al. 2020). We tested the FA approach with wild predators and the results are comparable to those obtained using SIs, the biotracer most widely used for ecological studies (Table 5).

**Table 5:** Proportions of diet estimated for leopard seals, using MixSIAR with fatty acid or stable isotope data

Sources	Diet estimation for leopard seals	
	Fatty acids	Stable isotopes
*A. gazella*	0.194 (0.084–0.275)	0.062 (0.002–0.232)
*E. superba*	0.663 (0.606–0.717)	0.632 (0.509–0.742)
*P. antarcticum*	0.111 (0.038–0.230)	0.077 (0.003–0.278)
*P. papua*	0.027 (0.006–0.050)	0.083 (0.004–0.250)
*Pareledone *sp	–	0.081 (0.002–0.300)
*M. ingens*	0.003 (0.000–0.012)	–

We do not expect to get identical diet predictions from models using SIs and FAs because the biotracers undergo different biochemical pathways and assimilate diet over potentially different time frames. In phocid seals, the SI in an entire vibrissae can represent diet assimilated for up to a year; where smaller sections of the vibrissae, can represent diet over days to weeks (Rogers et al. 2016). Rogers et al. (2016) measured the vibrissae growth and the rate of replacement of captive leopard seals and showed that vibrissae shed annually, and are lost asynchronously, thus, depending on when a vibrissae had been shed, it will represent growth (and so diet items assimilated) over weeks up to a year. Although the growth pattern of the vibrissae of the crabeater and Weddell seals are unknown, they are likely to represent diet assimilated over a similar timeframe to the leopard seal, as do other phocid seals (Hirons et al. 2001; Greaves et al. 2004; Newland et al. 2011; Beltran et al. 2015).

The timeframe integrated by blubber FAs will depend on the blubber section (layer) analysed. In most marine mammals, including crabeater (Guerrero and Rogers 2017), leopard (Guerrero et al. 2016) and Weddell seals (Wheatley et al. 2007), blubber FAs are stratified. This implies that FAs in the outer layer (the half closer to the skin) are in different proportions compared to the inner layer (the half closer to the muscle). The dietary signal in the inner layer more closely reflects dietary FAs, as they are preferentially stored in this section (Koopman 2007; Guerrero et al. 2016). Conversely, the outer blubber layer is usually more stable and does not seem to respond to short-term shifts in diet or body condition (Struntz et al. 2004). Outer layer FAs, for example, maintain their composition during lactation in Weddell seals (Wheatley et al. 2007), and northern elephant seals (Fowler et al. 2014). However, although the proportion of FAs may differ between layers, the overall FA pattern is similar (Hooker et al. 2001); thus, the outer layer is thought to be an indicator of longer-term diet (Moller et al. 2003; Budge et al. 2006; Guerrero et al. 2020) compared to the inner layer that reflects from hours to months (Kirsch et al. 2000; Budge et al. 2004, 2006). Here, we used only the outer layer for FA analysis since it can be obtained remotely, hence, requires comparatively less effort and lower economic costs than the collection of whole blubber samples. Therefore, although both vibrissae and outer blubber integrate diet history over long timeframes (potentially months to years), they may not represent the exact same timescale.

### Comparison of estimated diets

#### Crabeater seals

For the crabeater seal both SI and FA models identified krill as the main component of their diet, in agreement with previous studies (Laws 1977; Øritsland 1977; Green and Williams 1986; Lowry et al. 1988; Siniff 1991; Hückstädt et al. 2012; Botta et al. 2018). However, the FA model estimated krill to be a smaller contribution than the SI model (84% and 91%, respectively). Other studies based on SIs, have found krill to contribute 88% (Hückstädt et al. 2012) and 90% (Botta et al. 2018) to the diet of crabeater seals from the western Antarctic Peninsula. Similarly, studies based on conventional stomach and scat content analysis, which usually represent only summer diet, have found that crabeater seals can feed almost exclusively on krill (Laws 1977; Øritsland 1977), although the occasional occurrence of krill other than *E. superba*, octopus, and fish, in their food remains suggests that they display opportunistic feeding behaviour (Green and Williams 1986).

The slightly lower contribution of krill predicted by the FA model could be associated with an overestimation of sources containing higher proportions of fat. The lipid content of the fish species used as sources ranges from ~ 32 to ~ 38% of dry weight (Hagen et al. 2000; Stowasser et al. 2009) whereas krill may range from ~ 7% in winter to ~ 17% in summer (Virtue et al. 1993; Hagen et al. 2001). Since the outer blubber layer reflects a long-term diet, which probably integrates periods where krill lipids are reduced to a minimum during winter and early spring (Ikeda and Dixon 1982; Hagen et al. 1996), the fat stores of the consumer are potentially reflecting better those sources with higher lipid content.

Little is known about the fish species consumed by crabeater seals. Here we included the same prey species used by Hückstädt et al. (2012), which correspond to fish species known to be consumed by other krill specialists, the Antarctic fur seal and the Adélie penguin *Pygoscelis adeliae*. For crabeater seals, only the Antarctic silverfish *P. antarcticum* has been identified in food remains (Green and Williams 1986; Lowry et al. 1988); however, Green and Williams, (1986) found that whereas 4 out of 45 otoliths were from *P. antarcticum*, the majority (41) were from non-identified fish, suggesting that this is not the primary fish consumed by crabeater seals.

Unlike SIs, FAs were able to separate all fish species, indicating that consumption of the myctophid fish *E. antarctica* was more important than the other fish species. The SI model; however, attributed a minor contribution to the group Pa_Ea, composed by *E. antarctica* and *P. antarcticum*. However, without basal knowledge, it is difficult to determine whether the FA or the SI model provided a better prediction in this regard.

#### Weddell seals

Weddell seals feed primarily on fish (Siniff 1991); thus, we included several fish species that have been previously reported as their prey items. The FA model predicted that *P. antarcticum* was the most important item in their diet, whereas the SI model estimated that the group Pa_Ea_P; which included *P. antarcticum, E. antarctica* and the squid *Pareledone *sp; was the main component. Using SIs of blood and vibrissae of Weddell seals from the Ross Sea, Goetz et al. (2016) determined that the species group with the highest proportional contribution was that composed by *P. antarcticum* and *Trematomus newnesi*. Similarly, Botta et al. (2018) through SI analysis found that the fish group was the main contributor to the diet of Weddell seals from the western Antarctic Peninsula. Based on scat contents, Burns et al. (1998) found remains of *P. antarcticum* in 70–100% of the scats analysed, with little temporal variation over five years of study in McMurdo Sound. Plötz (1986) found that this fish constituted 61% and 94% of all otoliths, in two different years of study, and Casaux et al. (2006) found that *P. antarcticum* was within the first three predominant fish species by mass, in two different years, based on scat content analysis of Weddell seals from Cierva Point; the same location where our seals were sampled. Thus, we could infer that the FA model correctly identified the most important fish species in the diet, although we cannot confirm that proportions are accurate. Conversely, the SI model did not provide the same taxonomic resolution due to the isotopic similarity among sources, even when they belonged to different taxonomic groups (fish and cephalopod). This is common when using SIs; thus, other studies have not been able to identify prey items to a species level (e.g. Goetz et al. 2016; Botta et al. 2018).

Both approaches identified *G. nicholsi* as the second most important dietary item, with the same percentage of contribution (24%). According to scat content analyses of Weddell seals from the same study area (Casaux et al. 2006), *G. nicholsi* has a minor importance in their diet (between ~ 2 to ~ 4% importance by mass). There are other potentially important species that we did not include due to unavailability of either SI or FA data, such as the Antarctic cod *Dissostichus mawsoni* (Plötz 1986; Siniff 1991), *Trematomus* species (Plötz 1986; Plötz et al. 1991; Burns et al. 1998; Casaux et al. 2006), and some channichthyid species (Plötz 1986; Plötz et al. 1991; Casaux et al. 2006). Therefore, these results should be interpreted with caution.

Krill is known to have less importance in the diet of Weddell seals compared to crabeater and leopard seals (Forcada et al. 2012). Our results concord with the SI-based study of Botta et al. (2018) and other content analysis studies that found that, although present, krill was not relevant in the diet of the Weddell seals (Casaux et al. 1997, 2006).

#### Leopard seals

Leopard seals have the broadest diet of the Antarctic pack-ice seals, with krill, penguins, fish and other seals reported as the most frequent food items (Lowry et al. 1977; Øritsland 1977; Rogers and Bryden 1995; Krause and Rogers 2019; Krause et al. 2020). For leopard seals, we included the same five prey items for both FA and SI models. Krill was identified as the main component of the diet, and in similar proportions by the models that used the FA and SI data: 66% vs 63% respectively. Other studies in the same region also showed that krill was the most frequent and numerous prey found in leopard seal scats (Casaux et al. 2009), and according to SI data of vibrissae (Botta et al. 2018).

There are differences, however, between the models predictions of the importance of the other prey items. The model using SI data predicted an almost equal contribution of all other four sources, suggesting that leopard seals had a generalist diet. However, this could be due to the inability of the model to differentiate among prey items, since weakly informative data shifts the posterior distribution towards a generalist diet (i.e. the uninformative prior is really a generalist prior) (Stock and Semmens 2016). Conversely, FAs predicted a higher contribution of *A. gazella*, followed by *P. antarcticum*. The model using FA data potentially over represents the contribution of fur seals, since leopard seals prey on the fur seals between December and mid-February, when pups are 1–2 months old (Hiruki et al. 1999; Krause and Rogers 2019; Krause et al. 2020). Since the outer blubber reflects a long-term diet, the overall contribution of this prey species is expected to be smaller. Potentially, this is a limitation associated with the use of milk FAs as a proxy of pup FAs, since as seen in other pinniped species, blubber of pups does not exactly match that of the mother’s milk or blubber (Grahl-Nielsen et al. 2000; Wheatley et al. 2008). Similarly, milk SIs might not be the ideal proxy for pup SIs, since pup blood is enriched in δ^15^N and depleted in δ^13^C compared to milk (Cherel et al. 2015). However, in the absence of FA or SI data of Antarctic fur seal pups, this was the most representative solution.

### Implications for trophic studies

Our results demonstrate that the application of the Bayesian tool MixSIAR to FA data provides dietary estimations comparable to those obtained from SI data. Indeed, the use of both approaches has an increased economic cost due to laboratory analyses, which is an important limitation to the study. FAs have greater power of distinction among prey species, allowing higher taxonomic resolution. We, therefore, recommend the use of this complimentary approach especially when prey species are isotopically similar.

Because FAs and SIs produced similar results, the use of either approach is also recommended for dietary studies. In this regard, and since SIs are already being broadly used for quantitative ecological studies, we promote the use of FA data as an alternative. It has been demonstrated that FA data, using MixSIAR, correctly identifies the main dietary items of captive fish, seals and birds (Guerrero and Rogers 2020). We demonstrate that diet estimates of wild marine mammals do not differ from those obtained from SI data, in fact, can provide clearer results by identifying prey to a species level.

Unlike the SI approach, the accuracy of diet estimation based on FA data can be affected by prey lipid content. Fat-rich prey provides increased lipids for storage in the blubber; thus, diet estimation models based on blubber FAs can overestimate the contribution of prey with greater lipid content (Guerrero and Rogers 2020). We, therefore, recommend to determine lipid content for each prey species during the lipid extraction analysis. MixSIAR can account for prey lipid content by incorporating “concentration dependence” in the diet estimation model (Stock and Semmens 2013).

These analyses also demonstrate that the outer layer is a good proxy of long-term diet. It has been argued that the dietary signal is “erased” in the outer blubber layer and that inferences about diet should only been derived from the inner layer (Olsen and Grahl-Nielsen 2003). However, although the outer blubber has lower turnover rates than the inner layer (Budge et al. 2006), FAs from the diet are deposited into both layers, although in lower concentration in the outer section (Budge et al. 2004). Although more structural in nature (Budge et al. 2006; Guerrero and Rogers 2019), the outer blubber layer preserves valuable information of dietary history over long periods of time. This has important implications for field work, as the outer layer can be collected remotely, with minimum disturbance of the animal; making the use of blubber FAs more feasible for dietary studies. Additionally, this technique makes possible the simultaneous collection of, not only outer blubber, but skin and fur, which can be used for SI analysis.

Perhaps the weakest aspect of using these biotracers is how we account for trophic modification from source to consumer (Guerrero and Rogers 2020). Although controversial, the use of trophic discrimination derived from other consumer species is a common practice when using SIs quantitatively (e.g. Goetz et al. 2016; Sepúlveda et al. 2017). Since the quantitative use of FAs is more recent, only a few studies have used calibration coefficients derived from other consumer species (e.g. Thiemann et al. 2008). Rosen and Tollit (2012) warn against using calibration coefficients interchangeably, since they can be very species-specific. However, conducting feeding experiments to calculate the trophic modification for each study species is challenging, and for some species, not possible. Thus, the use of discrimination values derived from other species is a feasible alternative to estimate diets quantitatively.

Marine mammals integrate and reflect ecological variation across large spatial and long temporal scales (Moore 2008). Although their study is logistically challenging, new methodologies, like the approach we demonstrate here, allow us to generate quantitative data that is important to understand predator–prey dynamics on a long-term basis. Diet estimates of top predators are important to monitor ecosystem dynamics, as they can reflect changes occurring at lower trophic levels. Quantitative dietary data is also useful to understand conflicts between top predators and local fisheries, and in order to implement management programmes. Furthermore, dietary information is important to direct future research in wild populations and can be determinant to prioritise conservation.

## Supplementary Information

Below is the link to the electronic supplementary material.

Supplementary file1 (XLSX 37 KB)

## Data Availability

All data produced from this study are available in this manuscript and the supplementary material.

## Code availability

We used the MixSIAR code from Stock et al. (2018) which is available at https://github.com/brianstock/MixSIAR
